# Diagnostic Delay and Clinical Characteristics of Glomus Tumors in the Hand: A Case Series of 11 Patients

**DOI:** 10.7759/cureus.107434

**Published:** 2026-04-21

**Authors:** Hideaki Ishii, Takahiro Maeda, Misato Sakamoto, Tomoyasu Homma, Hiroyasu Ikegami, Shu Yoshizawa

**Affiliations:** 1 Department of Orthopaedic Surgery (Ohashi), Toho University, Tokyo, JPN

**Keywords:** diagnosis, glomus tumor, hand, magnetic resonance imaging, pain

## Abstract

Background: The aim of this study is to analyze the clinical characteristics of glomus tumors of the hand, with particular focus on diagnostic delay, tumor location, and the roles of physical examination and magnetic resonance imaging (MRI) in preoperative diagnosis.

Methods: This retrospective case series included 11 patients with histopathologically confirmed glomus tumors of the hand treated at a single institution between 2014 and 2021. Clinical characteristics and physical examination findings were evaluated in all patients. MRI findings and their concordance with pathological diagnosis were analyzed in the subset of patients who underwent preoperative MRI (n = 9). Analyses were primarily descriptive.

Results: The cohort consisted of eight women (73%) and three men (27%), with a mean age of 39.6 ± 10.3 years. Tumors were located in the subungual region in eight patients (73%) and in non-subungual regions in three patients (27%). The mean duration from symptom onset to diagnosis was 8.6 ± 6.3 years, with comparable durations between subungual and non-subungual tumors. Localized point tenderness was observed in 10 patients (91%), whereas cold hypersensitivity was present in four patients (36%), and the complete symptom triad was observed in four patients (36%).

Preoperative MRI was performed in nine patients and demonstrated a high lesion detection rate; however, accurate preoperative diagnosis was achieved in only six of nine patients (67%). None of the non-subungual tumors were correctly diagnosed before surgery. No single MRI sequence consistently provided optimal lesion visualization across cases. All patients underwent complete surgical excision, resulting in complete symptom resolution without recurrence.

Conclusions: Glomus tumors of the hand are associated with prolonged diagnostic delay regardless of tumor location. Although MRI is useful for detecting lesions, it is insufficient for establishing a definitive preoperative diagnosis. In contrast, localized point tenderness is a consistent and clinically valuable finding that may play a key role in raising diagnostic suspicion. These findings highlight the importance of integrating clinical symptoms, physical examination, and imaging to achieve accurate and timely diagnosis.

## Introduction

Glomus tumors are benign but clinically significant vascular neoplasms that arise from the glomus body, which is a specialized arteriovenous shunt involved in thermoregulation [1]. Although they account for only 1-5% of soft tissue tumors of the hand, glomus tumors are well recognized for causing severe, disproportionate pain relative to their size [1,2]. Despite their characteristic clinical features, glomus tumors are often misdiagnosed, resulting in a prolonged diagnostic delay [2-4].

The classic clinical presentation of glomus tumors consists of a triad of spontaneous pain, localized point tenderness, and cold hypersensitivity [1,3,4]. However, the complete triad is not consistently observed [4,5]. Diagnostic difficulty is further compounded by the tumors' small size and deep location within the nail bed or soft tissues of the hand [5]. Consequently, patients often undergo multiple evaluations before a definitive diagnosis is established [1].

Glomus tumors most commonly arise in the subungual region of the hand; however, they have also been reported in non-subungual locations, including the nail matrix, nail bed, fingertip pulp, as well as other regions of the hand outside the digits [1-5]. Tumors arising outside the subungual region often lack nail-related findings such as deformity or discoloration, which may make them more difficult to suspect based on physical examination alone. However, whether these non-subungual tumors tend to be associated with a longer diagnostic delay compared with subungual lesions has not been sufficiently investigated, and data regarding non-subungual glomus tumors remain limited.

Magnetic resonance imaging (MRI) is widely used to evaluate suspected glomus tumors [6-8]. Although MRI can facilitate the detection of small soft tissue lesions, its imaging findings are not specific and may overlap with those of other benign soft tissue tumors of the hand [1,4,6]. Consequently, accurate preoperative diagnosis is not always achieved even when a lesion is detected on MRI, particularly in tumors arising in atypical locations.

This retrospective study reviewed patients with hand glomus tumors treated at a single institution, focusing on diagnostic delay, tumor location including non-subungual sites, accuracy of preoperative diagnosis, and postoperative outcomes.

We hypothesized that non-subungual tumors are associated with a longer diagnostic delay and that clinical examination findings may be more reliable than MRI for diagnosis.

## Materials and methods

Study design

This retrospective case series was conducted at a single institution and included patients who underwent surgical treatment for glomus tumors of the hand between February 2014 and December 2021. Medical records were reviewed to identify eligible patients.

Inclusion and exclusion criteria

**Figure 1:**
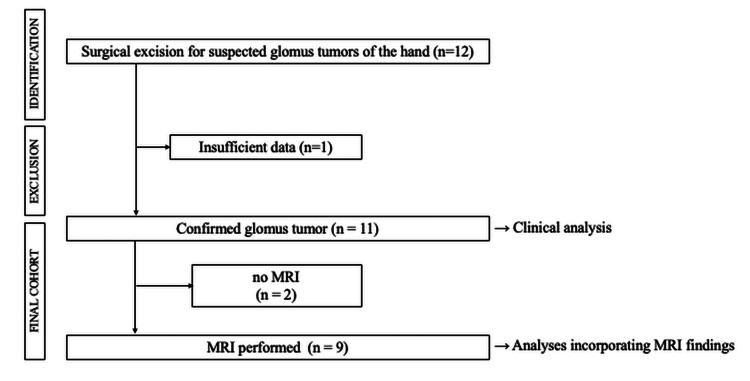
Study cohort. Twelve patients were identified, and 11 with confirmed glomus tumors were included after exclusion of one patient with insufficient data. Clinical analyses were performed in all patients, and analyses incorporating MRI findings were conducted in the nine patients who underwent preoperative MRI.

A total of 12 patients who underwent surgical excision for suspected glomus tumors of the hand were initially identified. Of these, one patient was excluded due to insufficient preoperative clinical data resulting from limited follow-up at our institution. Consequently, 11 patients with histopathologically confirmed glomus tumors were included in the clinical analysis. Among these, nine patients who underwent preoperative MRI were included in analyses incorporating MRI findings. The process of patient selection and inclusion is illustrated in the cohort flow diagram (Figure 1).

Ethical approval

The study protocol was approved by the institutional ethics committee, and the study was conducted in accordance with the principles of the Declaration of Helsinki. Patient anonymity and confidentiality were maintained throughout the study.

Data collection

Demographic data, including age and sex, were collected for each patient. Clinical information at presentation was reviewed, including the presence of spontaneous pain, localized point tenderness, cold hypersensitivity, and nail deformity. Point tenderness was assessed using Love’s test, a clinical maneuver involving localized pressure over the suspected lesion. Cold sensitivity was evaluated based on patient-reported symptoms in response to cold exposure. The duration of symptoms prior to diagnosis or surgical treatment was also recorded. Tumor location was determined based on intraoperative findings and categorized as subungual, fingertip pulp, or other regions of the hand. Ultrasonography was not systematically evaluated in this study and was therefore excluded from the analysis.

Clinical characteristics and physical examination findings were evaluated in all patients (n = 11). In the subset of patients who underwent preoperative MRI (n = 9), MRI studies were reviewed to assess tumor characteristics, including size, anatomical location, and signal intensity on T1-weighted, T2-weighted, and short tau inversion recovery (STIR) sequence, a fat-suppressed inversion recovery technique. MRI examinations were performed using a 3.0-T scanner. Imaging typically included T1-weighted, T2-weighted, and STIR sequences acquired in axial, coronal, and sagittal planes; however, not all sequences and planes were obtained in every case due to variations in clinical practice. Additionally, imaging parameters such as slice thickness were not standardized because of the retrospective study design. MRI findings were evaluated descriptively, with particular attention to features that may influence preoperative diagnostic accuracy. All patients underwent surgical excision of the tumor. Surgical outcomes, including perioperative complications, were documented. Postoperative follow-up data were reviewed to assess symptom resolution, residual pain or tenderness, and tumor recurrence.

Statistical analysis

Patients were categorized into two groups according to the concordance between the overall preoperative clinical diagnosis, based on clinical findings and imaging studies including MRI, and the postoperative pathological diagnosis. Clinical analyses were performed in all patients (n = 11). Analyses incorporating MRI findings, including the assessment of concordance between MRI-based diagnosis and postoperative pathological diagnosis, were conducted in the subset of patients who underwent preoperative MRI (n = 9). Due to the small sample size, analyses were primarily descriptive. Continuous variables were summarized as means with standard deviations or medians with ranges, as appropriate, and categorical variables were presented as frequencies and percentages. No formal hypothesis testing was performed. Statistical analyses were performed using IBM SPSS Statistics for Windows, Version 30 (Released 2024; IBM Corp., Armonk, New York, United States).

## Results

A total of 11 patients with surgically and histopathologically confirmed glomus tumors of the hand were included in the clinical analysis. The cohort consisted of eight women (73%) and three men (27%), with a mean age of 39.6 ± 10.3 years (range: 21-58) at the time of surgery. The mean follow-up period was 12.2 ± 13.1 months (range: 2-40). Tumor location, as confirmed intraoperatively, was subungual in eight patients (73%) and non-subungual in three patients (27%). Among the non-subungual cases, two (18%) were located in the fingertip pulp and one (9%) in the dorsum of the hand. With respect to digit involvement, tumors occurred in the thumb in three patients (27%), index finger in one patient (9%), middle finger in one patient (9%), ring finger in two patients (18%), little finger in three patients (27%), and outside the digits in one patient (9%).

The duration of symptoms prior to correct diagnosis varied widely among patients. The mean duration from symptom onset to diagnosis was 8.6 ± 6.3 years (range, 2-20). When stratified by tumor location, the mean duration was 9.0 ± 7.0 years (range: 2-20) in subungual tumors and 7.7 ± 5.0 years (range: 3-13) in non-subungual tumors. 

Patient demographics, clinical characteristics, postoperative outcomes, and individual symptom duration for each patient are summarized in Table 1.

**Table 1:** Clinical characteristics of the patients. Data are shown for individual patients. “+” indicates the presence of the symptom, and “−” indicates its absence. Diagnostic delay represents the interval from symptom onset to diagnosis.

Case	Sex	Age	Side	Digit	Location	Paroxysmal pain	Point tenderness	Cold Hypersensitivity	Previous surgery	Diagnostic Delay (years)	Recurrence
1	F	45	Left	Ring	Subungual	＋	＋	ー	No	20	None
2	M	58	Right	Hand	Dorsum manus	−	＋	ー	Yes	7	None
3	F	49	Left	Little	Subungual	＋	＋	ー	Yes	4	None
4	F	21	Left	Index	Pulp	＋	＋	＋	No	13	None
5	F	40	Right	Thumb	Subungual	＋	＋	ー	No	2	None
6	M	38	Right	Little	Pulp	−	−	ー	No	3	None
7	F	32	Left	Thumb	Subungual	＋	＋	＋	No	5	None
8	M	36	Left	Thumb	Subungual	＋	＋	＋	No	10	None
9	F	42	Left	Ring	Subungual	＋	＋	＋	No	2	None
10	F	47	Right	Little	Subungual	＋	＋	ー	Yes	11	None
11	F	28	Right	Middle	Subungual	＋	＋	ー	No	18	None

Clinical presentation varied among patients. Spontaneous pain was reported in nine patients (82%), whereas localized point tenderness was present in all patients except for one case with a subungual tumor of the thumb. No patients exhibited nail deformity, regardless of tumor location. Cold hypersensitivity was documented in four patients (36%). Overall, four patients (36%) exhibited the complete classic symptom triad. Among the three non-subungual cases, only one patient presented with the complete triad, whereas the remaining cases showed incomplete clinical features.

Preoperative MRI was performed in nine of the 11 patients and was used for further evaluation. Analyses incorporating MRI findings were therefore conducted in these nine patients. Tumor size and MRI signal characteristics varied among cases, and detailed MRI findings are summarized in Table 2.

**Table 2:** MRI findings and preoperative diagnosis. Data are shown for patients who underwent preoperative MRI (n = 9). Cases 8 and 11 did not undergo MRI and were therefore excluded. Signal intensity on MRI is described relative to surrounding soft tissue: “low” indicates hypointensity, “iso” indicates isointensity, and “high” indicates hyperintensity on each sequence. STIR indicates short tau inversion recovery, a fat-suppressed MRI sequence.

Case	Location	X-ray findings	MRI findings				Preoperative diagnosis	Pathological diagnosis
		Cortical erosion	Tumor size (mm)	T1	T2	STIR		
1	Subungual	None	3.7×3.3×2.4	low	high	high	Glomus	Glomus
2	Dorsum manus	None	11.1×8.0×9.8	iso	high	high	Leiomyoma / Epidermal cyst	Glomus
3	Subungual	None	0.9×1.2×1.0	iso	high	high	Glomus	Glomus
4	Pulp	None	2.5×1.4×2.0	low	iso	high	Schwannoma	Glomus
5	Subungual	None	2.0×2.9×1.8	low	high	high	Glomus	Glomus
6	Pulp	Yes	4.9×7.1×3.1	low	iso	high	Giant cell tumor	Glomus
7	Subungual	Yes	6.7×5.9×2.1	low	iso	high	Glomus	Glomus
9	Subungual	None	1.9×1.7×2.4	low	high	high	Glomus	Glomus
10	Subungual	Yes	4.7×2.1×4.1	low	iso	high	Glomus	Glomus

**Figure 2:**
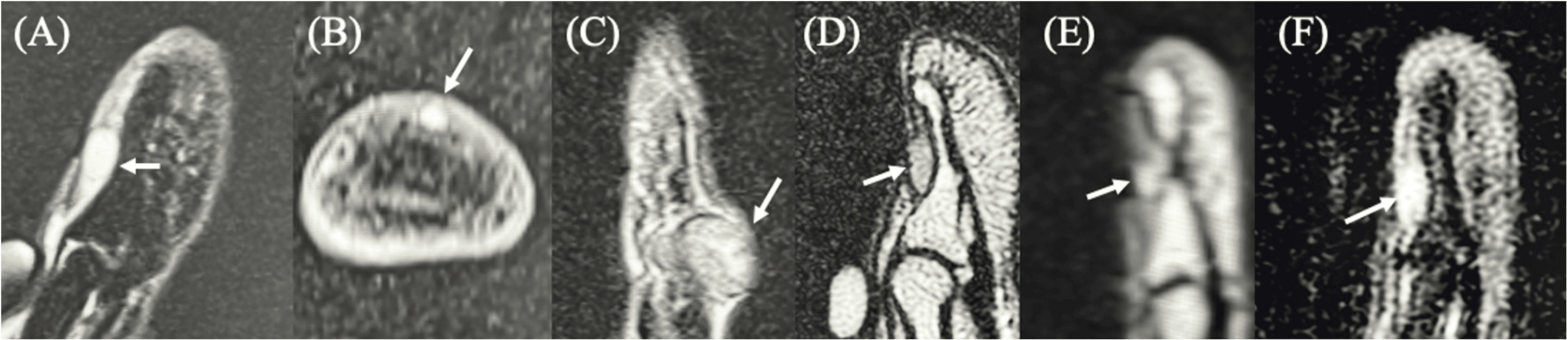
Representative MRI findings of subungual glomus tumors. Representative MRI findings of subungual glomus tumors in six patients who were correctly diagnosed preoperatively. Typical lesions are shown as small, well-defined nodules with low signal intensity on T1-weighted images and high or isointense signal intensity on T2-weighted and short tau inversion recovery (STIR) sequences. For each case, the image that most clearly demonstrated the lesion was selected from available MRI sequences and imaging planes. The optimal sequence and imaging plane for lesion visualization varied among cases. (A) T2-weighted, sagittal view, (B) T1-weighted, axial view, (C) T2-weighted, sagittal view, (D) T2-weighted, sagittal view, (E) T2-weighted, sagittal view, and (F) STIR, sagittal view.

In general, tumors exhibited low signal intensity on T1-weighted images and high or isointense signal intensity on T2-weighted and STIR sequences. However, no single MRI sequence consistently provided optimal lesion visualization across all cases. The sequences and imaging planes that best demonstrated the tumor varied among patients, with some lesions more clearly identified on T1-weighted, T2-weighted, or STIR images. Among the nine patients who underwent preoperative MRI, six were correctly diagnosed with glomus tumors based on an integrated assessment of imaging findings, clinical symptoms, and physical examination. All of these correctly diagnosed cases were subungual tumors, whereas none of the non-subungual tumors were accurately diagnosed preoperatively. Representative MRI findings of subungual tumors in these six cases are shown in Figure 2. These cases were selected to illustrate typical imaging features of subungual glomus tumors. Notably, the sequences and imaging planes that most clearly demonstrated the lesions differed among cases, further highlighting the variability in MRI visualization.

Among the three patients with non-subungual tumors, none were correctly diagnosed as glomus tumors preoperatively. Instead, alternative diagnoses were suggested based on imaging findings in all cases, including leiomyoma or epidermal cyst, neurilemmoma, and giant cell tumor of the tendon sheath.

These cases illustrate the diagnostic challenges associated with glomus tumors arising in non-subungual locations, where clinical presentation and imaging findings may be less characteristic. The clinical features and imaging findings of these representative cases are described below.

Case presentation

Case 2

A 58-year-old male, seven years prior to presenting to our clinic, underwent surgery at an outside institution for a mass located on the dorsum of the index metacarpophalangeal joint. One month after the initial surgery, he developed persistent pain on the ulnar side of the excised area suggesting recurrence but received no further treatment for several years. 

**Figure 3:**
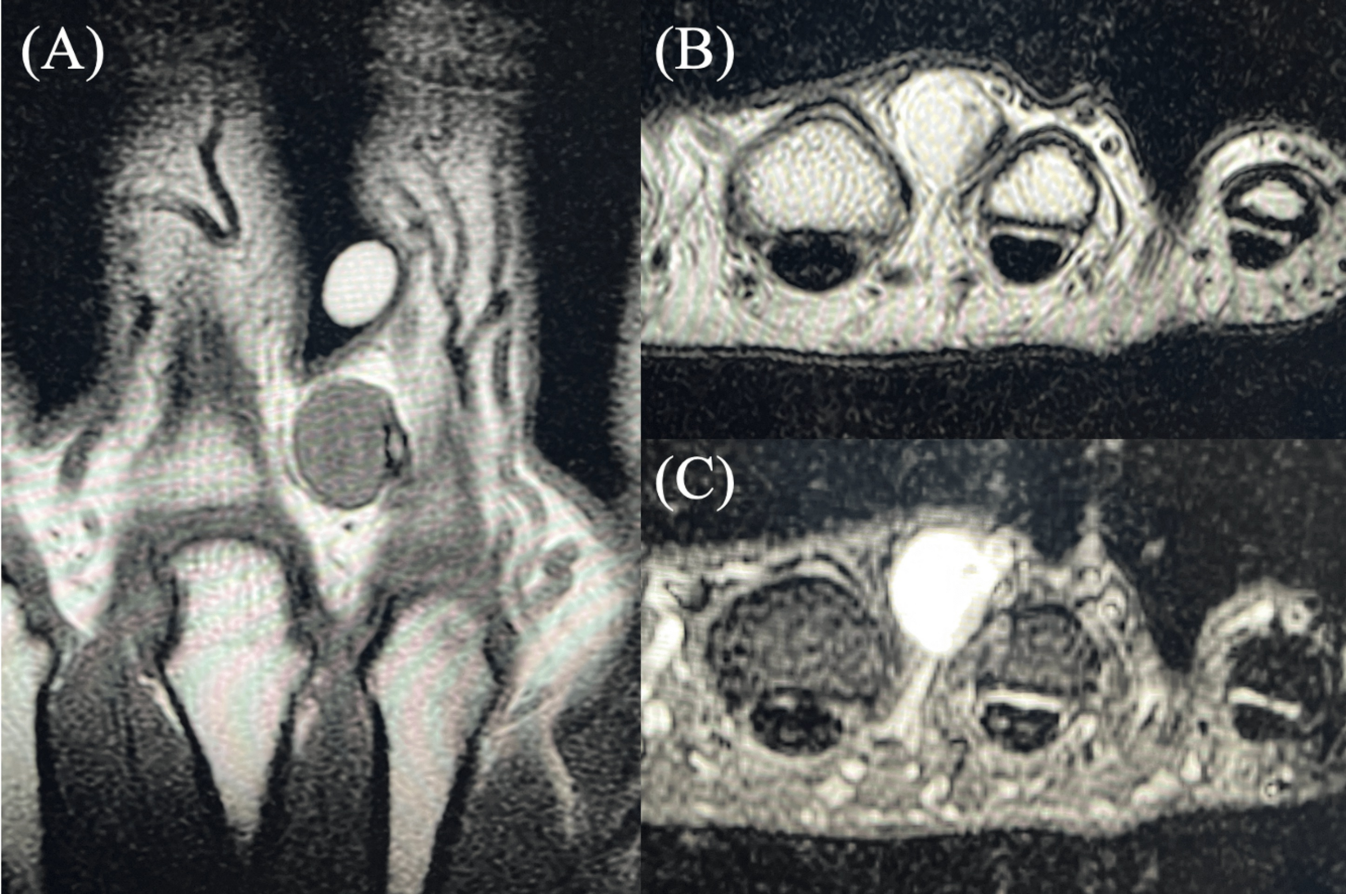
Preoperative MRI findings for case 2. A tumor with well-defined borders is present between the index and middle fingers. (A) Isointense signal on a coronal T1-weighted image. (B) High signal intensity on an axial T2-weighted image. (C) High signal intensity on an axial STIR image. STIR: Short tau inversion recovery

Preoperative MRI performed at our institution revealed a tumor mass measuring 11.1 × 8.0 × 9.8 mm between the index and middle finger dorsum. MRI findings showed a heterogeneously isointense signal on T1-weighted (T1W) images and a high signal intensity on T2-weighted (T2W) and STIR sequences (Figure 3). Clinically, the patient reported tenderness but no strong spontaneous pain or cold sensitivity. Based on the MRI findings, the radiologist's presumptive diagnoses included leiomyoma or epidermal cyst, excluding the glomus tumor from the initial differential diagnosis.

Case 4

**Figure 4:**
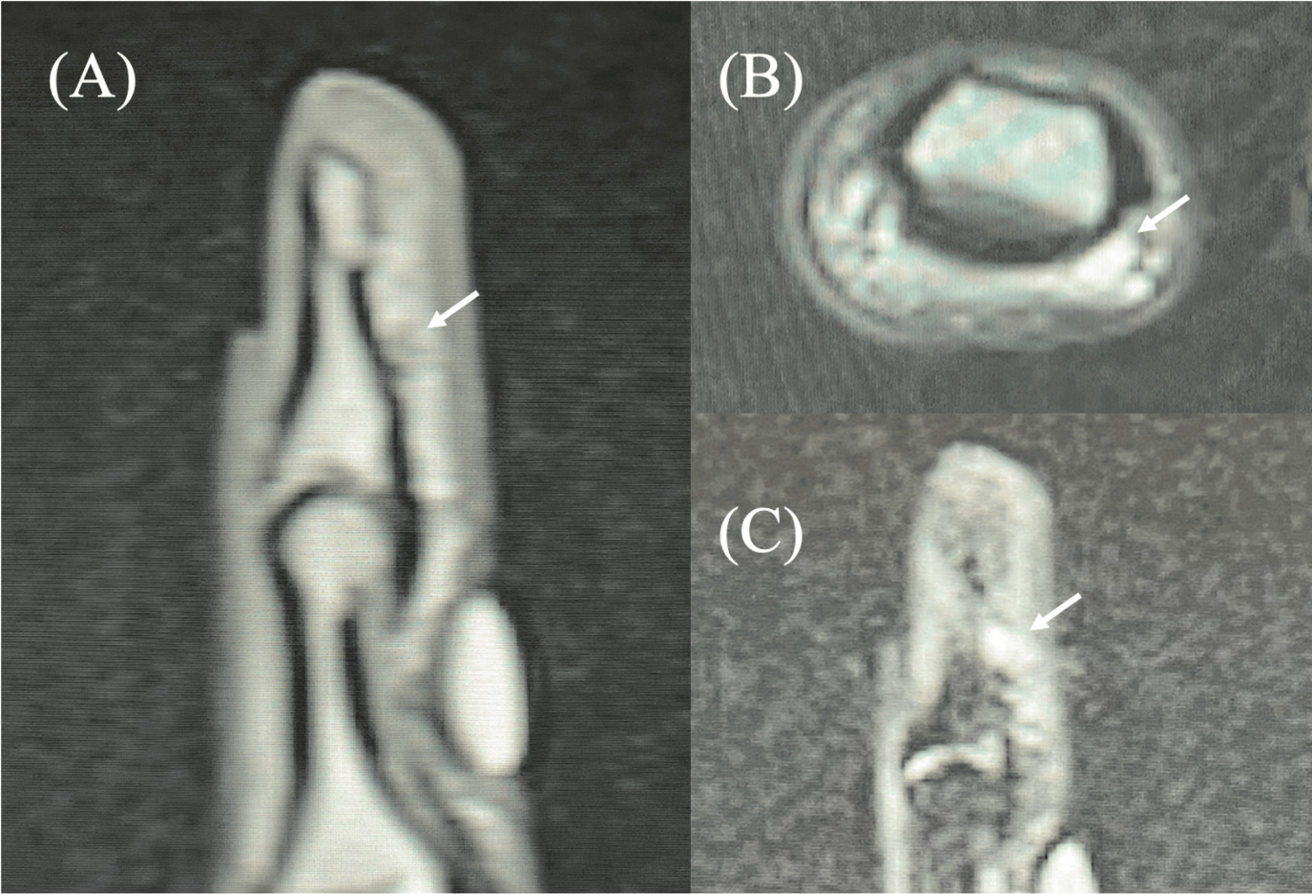
Preoperative MRI findings for case 4. A small nodular lesion is visible on the fingertip. (A) Low signal on a sagittal T1-weighted image. (B) Isointense signal on an axial T2-weighted image. (C) High signal intensity on a sagittal STIR image. STIR: Short tau inversion recovery

A 21-year-old female patient had suffered from chronic pain in the pulp of the index finger for 13 years. During this time, the patient had visited several medical institutions but had not received a definitive diagnosis. Clinically, the patient exhibited spontaneous pain and point tenderness, along with a mild exacerbation of symptoms upon cold stimulation. The patient reported that direct compression caused the most severe pain, disabling her ability to type during desk work. MRI revealed a small mass lesion measuring 1.4 × 2 × 2.5 mm in the distal index finger pulp, correlating precisely with the area of tenderness (Figure 4). The mass showed low signal intensity on T1W, isointense signal on T2W, and high signal intensity on STIR sequences. Preoperative MRI reading by the radiologist diagnosed the lesion as a neurilemmoma.

Case 6

**Figure 5:**
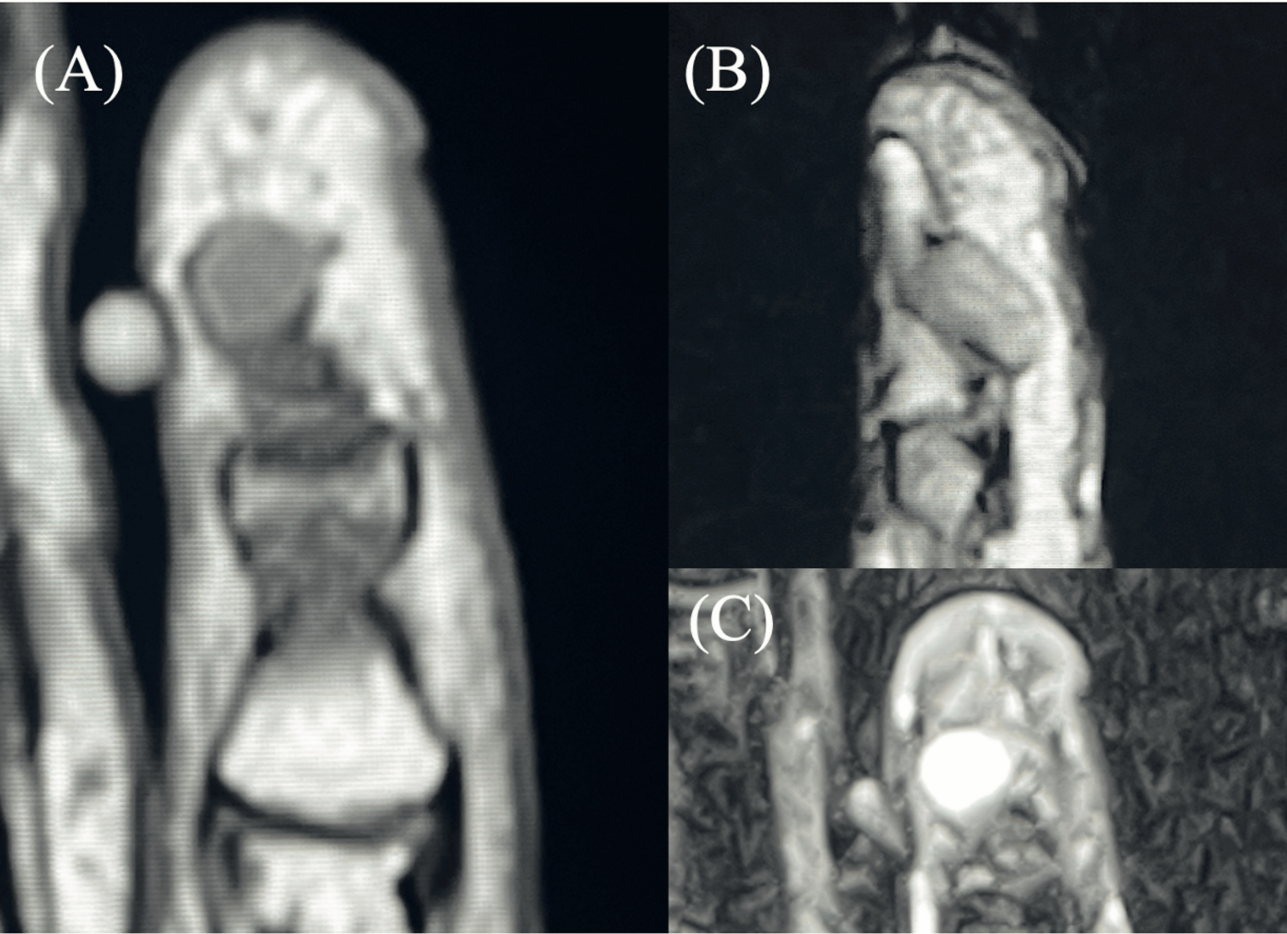
Preoperative MRI findings for case 6. A tumorous lesion that compresses the distal phalanx of the fingertip exists. (A) Low signal on a coronal T1-weighted image. (B) Isointense signal on a sagittal T2-weighted image. (C) High signal intensity on an axial STIR image. STIR: Short tau inversion recovery

A 38-year-old male patient recognized a persistent pain in the pulp of the little finger for three years. Preoperative X-ray showed an osseous defect in the distal phalanx. In contrast to the initial presentation, a clinical examination revealed only point tenderness in the finger pulp. The patient did not exhibit spontaneous pain or cold hypersensitivity. The MRI scan revealed a mass lesion in the pulp measuring 4.9 × 7.1 × 3.1 mm (Figure 5). The tumor displayed low signal intensity on T1W images, isointense signal on T2W images, and high signal intensity on STIR sequences. The radiologist's preoperative MRI diagnosis was a giant cell tumor of the tendon sheath.

All patients underwent complete surgical excision of the tumor. Histopathological examination confirmed a benign glomus tumor in all cases. Postoperatively, complete resolution of symptoms was achieved in all patients, and no recurrence was observed during the follow-up period.

## Discussion

Glomus tumors of the hand are well recognized for their diagnostic difficulty and the frequent delay between symptom onset and definitive treatment. Previous studies have reported prolonged diagnostic delays, often extending over several years, largely due to the small size of the tumor and its nonspecific clinical presentation [3,4,7,9]. In the present study, a similarly prolonged delay was observed, with a mean duration of 8.6 years, indicating that delayed diagnosis remains a persistent clinical problem.

Glomus tumors most commonly arise in the subungual region [1,4,9]. In the present study, no clear difference in diagnostic delay was observed between subungual and non-subungual tumors. The duration of symptoms was comparable between the two groups; however, this finding should be interpreted with caution, given the small sample size and the descriptive nature of the analysis. These results suggest that factors other than tumor location, including clinical presentation, may influence the time to diagnosis.

MRI is commonly used in the evaluation of suspected glomus tumors and facilitates the detection and localization of small soft tissue lesions [3,6,7]. Its usefulness extends beyond typical subungual lesions, as it can detect tumors in non-subungual or atypical locations and may also aid in screening for multifocal disease. In addition, MRI provides important information regarding tumor size, depth, and precise anatomical location, thereby contributing to accurate localization and surgical planning [7,8]. However, MRI findings are not always specific to glomus tumors, and the characteristic signal patterns may overlap with those of other benign soft tissue tumors of the hand [6,7]. Furthermore, small lesions or tumors in certain anatomical locations may not be detected, resulting in false-negative findings despite clinically significant symptoms [3,5]. In our series, although MRI demonstrated a high detection rate, accurate preoperative diagnosis was not always achieved. Notably, none of the non-subungual tumors were correctly diagnosed before surgery, despite lesion detection in most cases.

These findings highlight the limitations of relying solely on imaging studies for diagnosis. While MRI provides valuable anatomical information, it is not sufficient to establish a definitive diagnosis. The variability in optimal MRI sequences observed in this study further underscores the limitations of relying on imaging alone. In contrast, localized point tenderness was observed in most patients and represented the most consistent clinical finding in this cohort, suggesting that physical examination may play an important role in raising diagnostic suspicion, particularly in atypical presentations. However, these observations are based on a small cohort and were not formally compared using statistical analyses and therefore should be interpreted with caution. Taken together, these findings indicate that accurate diagnosis of glomus tumors requires integration of clinical symptoms, physical examination findings, and imaging information, rather than reliance on a single diagnostic modality.

It should also be noted that MRI was performed in most patients in this study, likely reflecting the accessibility of advanced imaging modalities in the Japanese healthcare setting. This may have influenced the diagnostic process and should be considered when interpreting the role of MRI in clinical practice. Ultrasonography has been reported as a useful, non-invasive imaging modality for superficial soft tissue tumors, including glomus tumors [5]. However, it was not systematically evaluated in this study. In addition, ultrasonography alone may be insufficient for definitive diagnosis, particularly for small or subungual lesions.

The classic symptom triad of spontaneous pain, localized point tenderness, and cold hypersensitivity has traditionally been considered characteristic of glomus tumors [1,3,4,9]. However, this triad is not universally present [1,10]. In our series, only four patients exhibited the complete triad, and only one non-subungual case demonstrated all three symptoms.

Consistent with previous reports indicating that localized point tenderness is observed more frequently than cold hypersensitivity [10], localized point tenderness was the most consistently observed clinical finding in our cohort. This reinforces its diagnostic importance in both typical and atypical presentations. Notably, localized point tenderness was present in 10 of 11 patients (91%), whereas accurate preoperative diagnosis based on integrated clinical and imaging assessment was achieved in only 6 of 11 patients (55%). Although these observations were derived from a small cohort and were not subjected to formal statistical comparison, they suggest that characteristic physical examination findings-particularly localized point tenderness-may play an important role in raising diagnostic suspicion.

Complete surgical excision remains the definitive treatment for glomus tumors and is associated with excellent outcomes [9]. In our series, all patients experienced complete symptom resolution following excision, with no recurrence observed during follow-up. These findings are consistent with previous reports demonstrating that surgical excision is both diagnostic and curative.

Although glomus tumors are generally benign, malignant transformation is rare. Malignant glomus tumors and tumors of uncertain malignant potential have been reported infrequently and are often associated with larger size and deeper location [11]. While uncommon, these findings underscore the importance of timely diagnosis and treatment.

This study has several limitations. First, its retrospective design, small sample size, and single-institution setting may limit the generalizability of the findings. Second, MRI parameters, including slice thickness, were not standardized due to the retrospective nature of the study. Third, cold sensitivity was assessed based on patient-reported symptoms without a standardized testing protocol. Fourth, interobserver variability in MRI interpretation was not evaluated. Fifth, statistical analyses were primarily descriptive, and no formal statistical comparisons were performed due to the limited sample size. Finally, potential diagnostic bias cannot be excluded, as clinical diagnoses were based on routine clinical practice. Nevertheless, the consistent clinical patterns observed, particularly regarding the diagnostic value of physical examination and the limitations of imaging, support the clinical relevance of our findings.

## Conclusions

Glomus tumors of the hand remain challenging to diagnose, particularly when arising in non-subungual locations. In this study, a prolonged diagnostic delay was observed, and no clear difference in diagnostic delay was identified between subungual and non-subungual tumors; however, this finding should be interpreted with caution given the small sample size. Although MRI was useful for detecting lesions, it was not sufficient for establishing a definitive preoperative diagnosis. In contrast, localized point tenderness was consistently observed and appeared to play an important role in raising clinical suspicion.

Clinicians should maintain a high index of suspicion for glomus tumors in patients presenting with persistent, localized hand pain and point tenderness, regardless of tumor location. Early recognition and prompt surgical excision are essential to relieve symptoms and achieve optimal clinical outcomes.
